# Draft genome sequence data of *Streptomyces antibioticus* SCBA4 isolated from the Soils of Surigao del Sur, Philippines

**DOI:** 10.1016/j.dib.2026.113203

**Published:** 2026-08-27

**Authors:** Cherray Gabrielle A. Macabecha, Irene A. Papa

**Affiliations:** a Agricultural Systems Institute, College of Agriculture and Food Science, University of the Philippines Los Baños, Philippines; b National Institute of Molecular Biology and Biotechnology (BIOTECH), University of the Philippines Los Baños, Philippines

**Keywords:** Secondary metabolites, *Streptomyces antibioticus* SCBA4, antibiotics, Actinobacteria, Natural products

## Abstract

The draft genome of *Streptomyces antibioticus* SCBA4 isolated from the soils of Surigao del Sur, Philippines is reported here. *S. antibioticus* is a member of the phylum Actinomycetota, a diverse group of Gram positive, high G+C content bacteria well known for their production of a variety of bioactive compounds. Sequencing using the Illumina NovaSeq 6000 platform yielded a 8,616,999 bp genome across 36 contigs with 7621 coding sequences, 87 tRNA, and 1 tmRNA. Consistent with the members of the same phylum, the GC content was 71.83% and was found to contain putative gene clusters of secondary metabolites such as NRPs, terpenes and polyketides. The genome sequence has been deposited at NCBI under the accession number JBSWZT020000000.

Specifications Table

**Table:** 

Subject	Biology
Specific subject area	Microbiology, Bacterial Genomics
Type of data	Table, Figure, Analyzed
Data collection	Genomic DNA was extracted using ZR Fungal/Bacterial DNA MiniPrep Kit (Zymo Research, Irvine, CA) and sequenced using the Illumina NovaSeq 6000 platform with 150-bp paired-end setting. After trimming using Trim Galore version 0.6.7 (Babraman Bioinformatics), QC reads were assembled de novo using SPAdes V3.13.0 and polished using Pilon version 1.23⁠, implemented in Unicycler 0.5⁠. De Novo assembled contigs (David et al 2020). Assembly quality was assessed with BUSCO v5.8.3 (Manni et al 2021) using the s*treptomyces*_odb12 database. Gene calling and annotation is done using Prokka V1.14.6 through GALAXY Europe (usegalaxy.eu).
Data source location	*Streptomyces antibioticus* SCBA4 was isolated from the soils of Surigao del Sur, Philippines. The isolates are kept in glycerol stocks at -20°C at the National Institute of Molecular Biology and Biotechnology (BIOTECH)- University of the Philippines Los Baños
Data accessibility	This Whole Genome Shotgun project has been deposited at DDBJ/ENA/GenBank under the accession JBSWZT000000000. The version described in this paper is version JBSWZT020000000. Repository name: NCBI (National Center for Biotechnology Information) Data identification number: BioProject accession number PRJNA1368391 Direct URL to data: http://www.ncbi.nlm.nih.gov/bioproject/1368391
Related research article	none

## 1 Value of the Data

• The draft genome of *Streptomyces antibioticus* SCBA4 may be useful in providing insights about the ecology and taxonomy of actinobacteria especially in the Philippines and Southeast Asian region.
• The genomic data presented in this study expands the available genetic resources for drug discovery while contributing to the understanding of microbial diversity in the Philippines, where bacterial genomic resources remain significantly underrepresented.
• Researchers working on environmental microbiology, genomics, and biotechnology from soil actinobacteria may find value in this genomic data in investigating natural products and bioactive compounds such as antimicrobials and antitumors.
• The genome sequence data may be used in comparative genomic analysis of *Streptomyces* species and contributes to the repository of actinobacterial genomes that produce a wide variety of bioactive compounds.

## 2 Background

The members of the genus *Streptomyces* are well known producers of a wide variety of bioactive secondary metabolites with numerous clinically and agriculturally important compounds, including antibiotics, antifungals, anticancer agents, and herbicides. However, the increasing concern for the fast growth in resistance to antibiotics, cancer therapeutics, and pesticides, coupled with the decline in natural products discovery, calls for the urgent need to identify novel bioactive compounds. Over the last decade, environments like desert soils, mountains, icy places and marine environments were found to harbor new *Streptomyces* strains which could also contain unexplored cryptic or silent biosynthetic gene clusters. Consequently, contemporary discovery strategies utilize genome mining, metagenomics, and bioinformatics to accelerate discovery of new bioactive natural compounds from these microbial reservoirs[1]. *Streptomyces antibioticus* is a soil borne actinobacteria with antimicrobial activity against numerous pathogens, with a great potential against multi-drug resistant pathogens like methicillin-resistant *Staphylococcus aureus* and vancomycin-resistant *Enterococcus*. Beyond this, continued investigation of *S. antibioticus* strains from different environment is a good opportunity for the discovery of novel natural products for pharmaceutical and biotechnological applications[2]. This work reports the draft genome of *Streptomyces antibioticus* SCBA4 isolated from the agricultural soils of Surigao del Sur, Philippines.

## 3 Data Description

**Fig. 1:**
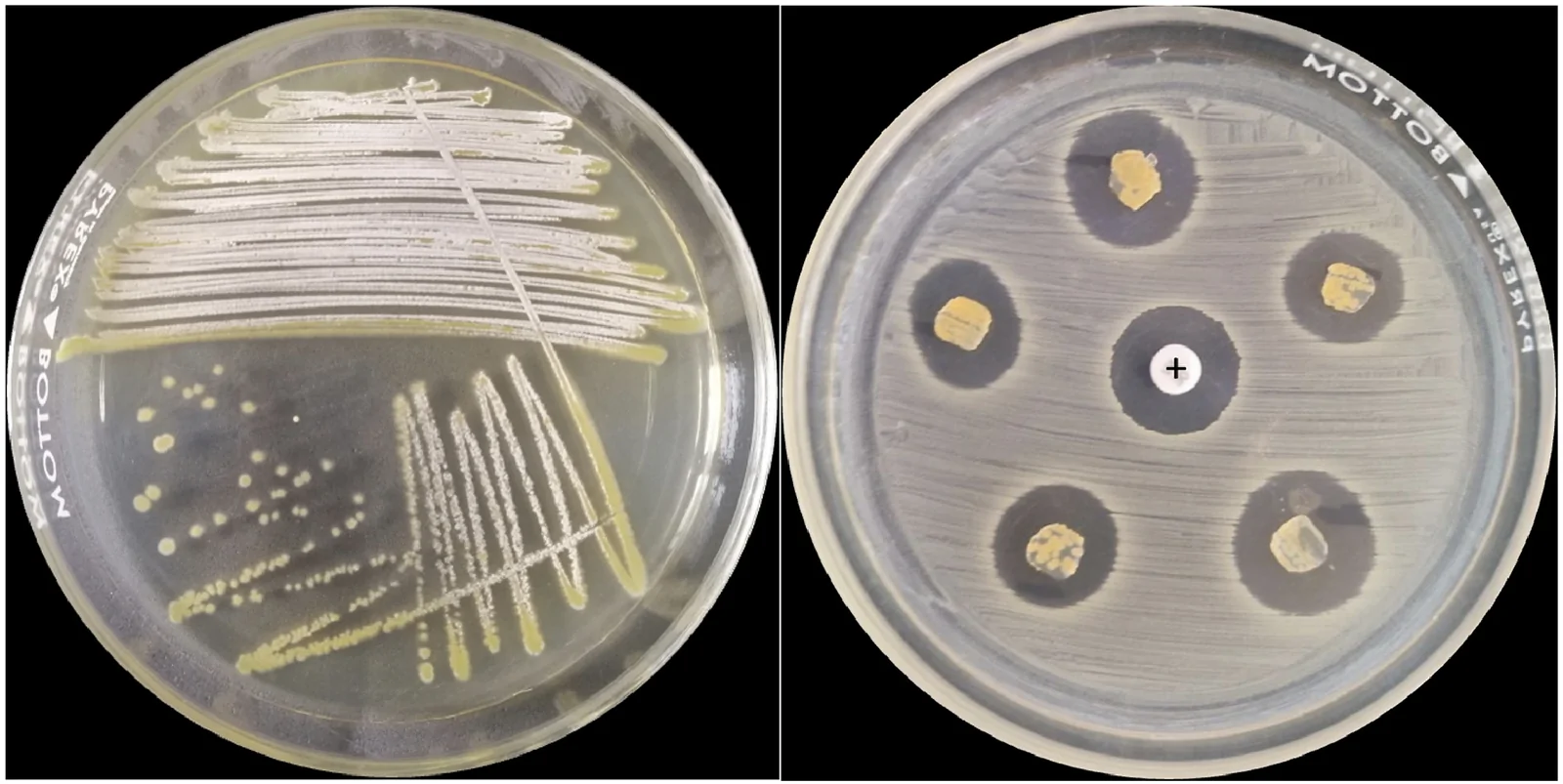
Colony morphology and growth of *S. antibioticus* SCBA4 on YMA after 3 days at 28°C (left) and representative plate showing antimicrobial activity against MRSA BIOTECH 10378 (right) with vancomycin as the positive control.

*Streptomyces antibioticus* SCBA4 was isolated from agricultural soils from Surigao del Sur, Philippines. The isolate exhibited moderate growth of yellow to powdery white colonies on Yeast Malt Agar (YMA) after 3 days at 28°C. Margins are initially entire but turn fuzzy after 3 days, with the white powdery texture starting in sections where there is more growth. Preliminary antimicrobial assay against methicillin-resistant *Staphylococcus aureus* BIOTECH 10378 showed an average zone of inhibition of 17.08 cm (Fig. 1). The genome features are summarized in Table 1. The draft genome was assembled into 36 contigs yielding a total genome length of 8,616,999 bp, N50 value of 521,904, and L50 value of 5. *S. antibioticus* SCBA4 has a high GC content of 71.83% characteristic of other members of the genus. Genome annotation showed a total of 7,621 CDS, 87 tRNAs, and 1 tmRNA. Although the genome assembly yielded 36 contigs, it was found to be mostly complete with 99.7% complete BUSCOs consisting of 99.6% single copy and 0.1% duplicated orthologs. This confirms that the primary metabolic and biosynthetic pathways were successfully captured, providing a reliable genomic blueprint for further functional studies. antiSMASH revealed 10 out of the 36 regions with putative gene clusters involved in the synthesis of secondary metabolites including those with antibiotic and antitumor activities [[3], [4]]. A total of 27 BGCs comprising diverse classes of secondary metabolite pathways were detected, predominantly nonribosomal peptide synthetases (NRPS) and polyketide synthases (PKS) as summarized in Table 2. Ten (10) BGCs exhibited high similarity to previously characterized clusters, including those predicted to the biosynthesis of actinomycin D, scabichelin, antimycin A1B, and ε-Poly-L-lysine, hopene, albaflavenone and isorenieratene, and other compounds like y-butyrolactone, and ectoine. BGCs in *S. antibioticus* SCBA4 also has high similarity confidence with some PKS like fredericamycin A. These BGCs encompass pathways associated with antimicrobial (actinomycin D, antimycin A1B) and antitumor (kanamycin, fredericamycin A) compounds, siderophores (scabichelin), terpenoids (hopene, albaflavenone) and signaling molecules (γ-butyrolactone). Additionally, a number of core genes encode for hypothetical proteins with no functional assignments, which may represent novel biosynthetic genes. Collectively, the results reveal the biosynthetic capability of *S. antibioticus* SCBA4, highlights its potential as a source of natural products relevant to medicine, agriculture, and biotechnology, and suggests that this isolate may harbor undiscovered pathways capable of producing novel natural products.

**Table 1:** Genomic Features of *S. antibioticus* SCBA4.

Features	Count
Contigs	36
Total length	8616999
N50	521904
N90	173775
auN	751888
L50	5
L90	17
GC (%)	71.83
# N's per 100 kbp	0
# N's	0
CDS	7621
Genes	7709
tRNA	87
tmRNA	1

**Table 2:** Putative gene clusters for secondary metabolites in SCBA4.

Type	Similarity Confidence	Cluster Size (bp)	Most similar known cluster	Core Gene/s
NRPS	High	60,558	actinomycin D	*dhbF, dltA_1*
NRPS-like	–	43,921	–	*dltA_2*
terpene-precursor, NRPS	High	61,176	scabichelin	HEPNBAOA_00405, *dltA_3*
RiPP-like	Low	10,216	informatipeptin	HEPNBAOA_00568
hglE-KS, lanthipeptide-class-i, T1PKS	–	51,479	–	HEPNBAOA_00775*, nisC, pcm_1,* HEPNBAOA_00779, HEPNBAOA_00780
terpene	High	26,725	hopene	*shc,* HEPNBAOA_00848, *crtB_1, hpnC*
hydrogen-cyanide, indole	Low	23,530	Azodyrecin A/Azodyrecin B/Azodyrecin C	*hcnC_1, hcnA, hcnB,* HEPNBAOA_00979
T1PKS,NRPS	High	52,571	antimycin A1B	HEPNBAOA_01092, *dltA_4*
NI-siderophore	–	31,280	–	HEPNBAOA_01182, HEPNBAOA_01183
terpene, butyrolactone	High	22,190	γ-butyrolactone	*cyc2,* HEPNBAOA_01325
RiPP-like	–	11,326	–	HEPNBAOA_01366
NI-siderophore	Low	29,956	kinamycin	*iucA*
T2PKS, T1PKS, NRPS-like, terpene-precursor	High	131,143	spore pigment	HEPNBAOA_01774, HEPNBAOA_01775, HEPNBAOA_01787, *fadA_3, pikAI, menE_3,* HEPNBAOA_01827
NRPS	Low	44,278	SF2768	*dltA_5*
T1PKS	–	53,578	–	*fadA_4*
terpene, melanin	Medium	36,048	melanin	HEPNBAOA_02223, HEPNBAOA_02228, HEPNBAOA_02237
furan, butyrolactone	–	23,797	–	HEPNBAOA_03027, HEPNBAOA_03036
T2PKS	High	72,495	fredericamycin A	HEPNBAOA_03663, HEPNBAOA_03664
NI-siderophore	Medium	29,770	FW0622	*iucC*
melanin	–	10,507	–	HEPNBAOA_04285
NAPAA	High	33,867	ε-Poly-L-lysine	*dltA_6*
terpene-precursor	Low	21,125	echoside A/echoside B/echoside C/echoside D/echoside E	HEPNBAOA_05541
terpene	High	21,053	albaflavenone	*cyc1*
lanthipeptide-class-iii, terpene	High	35,632	isorenieratene	*ramC,* HEPNBAOA_05643, HEPNBAOA_05645, *crtB_2*
RiPP-like	–	10,825	–	*lin*
azole-containing-RiPP	–	23,407	–	HEPNBAOA_06008, HEPNBAOA_06010
ectoine	High	10,405	ectoine	*ectC*

Annotation of carbohydrate-active enzymes using dbCAN3 identified 487 putative CAZymes, of which 270 were considered high-confidence annotations based on support from at least two of the three prediction methods (HMMER, DIAMOND, and/or dbCAN-sub) as summarized in Table 3. Glycoside hydrolases (GHs) represented the largest group (106 genes, 39.3%), followed by glycosyltransferases (GTs; 59 genes; 21.9%) and carbohydrate-binding modules (CBMs; 27 genes; 10%). Predominance of glycoside hydrolases indicates a strong capacity to degrade complex polysaccharides from plant residues which suggests an important role in the decomposition of soil organic matter. Abundance of GTs shows that *S. antibioticus* SCBA4 can synthesize a variety of polysaccharides and CBMs enhance its carbohydrate degradation efficiency by promoting enzyme binding to substrates like cellulose and chitin [4]. Additional CAZyme categories include carbohydrate esterases (CEs; 23 genes; 8.5%), which also aid in breakdown of plant cell wall polysaccharides. Auxiliary activity enzymes (AAs, 13 genes), and polysaccharide lyases (PLs, 6 genes) in *S. antibioticus* SCBA4 also aid in the breakdown of other polysaccharides like pectin and lignin . Thirty-six (36) genes were assigned as other CAZyme-related proteins. The collection of CAZymes in *S. antibioticus* SCBA4 indicates this isolate’s ability to utilize a number complex polysaccharides, reflecting its ecological role in soil organic matter decomposition and nutrient cycling [5]. Together with the diverse biosynthetic gene clusters identified using antiSMASH, the metabolic capabilities exhibited by *S. antibioticus* SCBA4 is characteristic of soil actinobacteria which are well-known for their ability to produce a wide variety bioactive secondary metabolites while contributing to organic matter turnover, nutrient transformation, and microbial interaction in soil environments.

**Table 3:** CAZyme class distribution of *Streptomyces antibioticus* SCBA4.

CAZy class	Number of genes	Percentage (%)
Glycoside hydrolases (GH)	106	39.3
Glycosyltransferases (GT)	59	21.9
Other CAZymes	36	13.3
Carbohydrate-binding modules (CBM)	27	10.0
Carbohydrate esterases (CE)	23	8.5
Auxiliary activities (AA)	13	4.8
Polysaccharide lyases (PL)	6	2.2
Total	270	100

### 3.1 Phylogenetic analysis

**Fig. 2:**
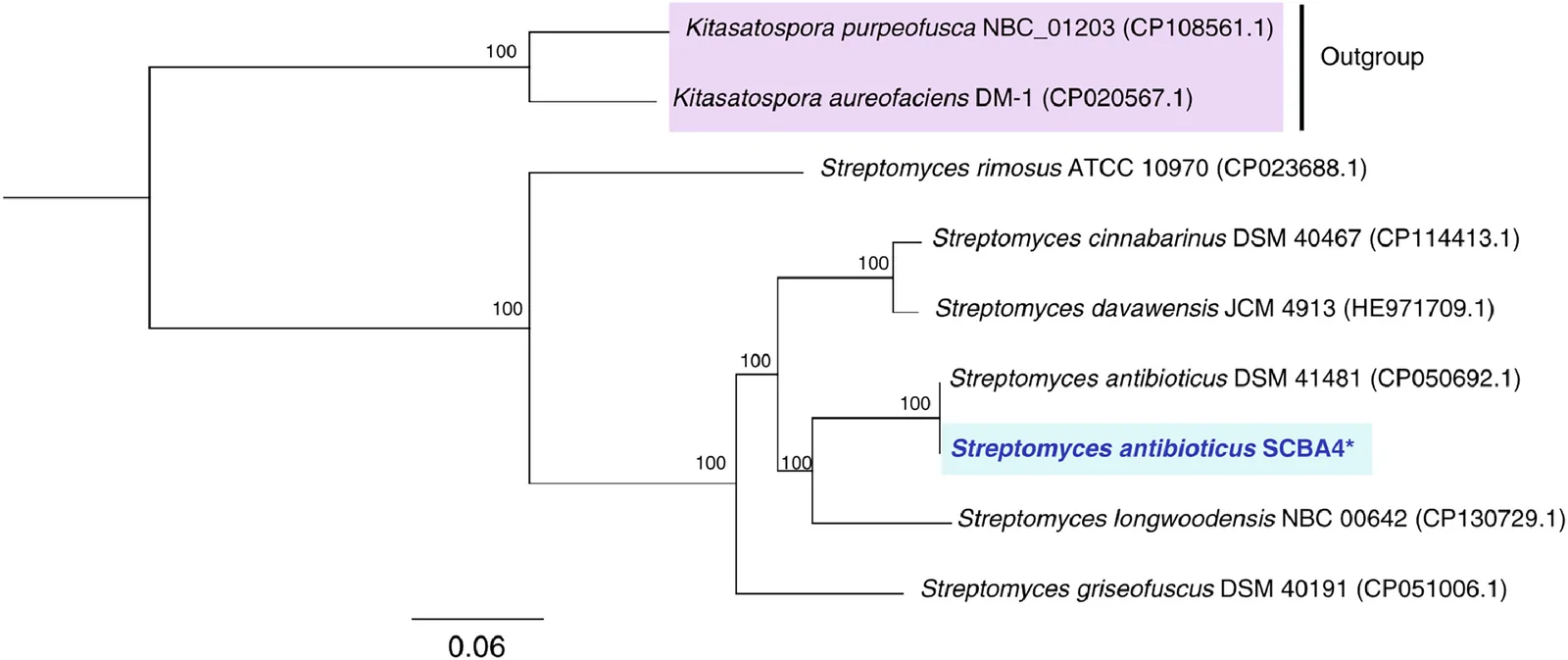
Maximum likelihood tree generated from using IQTree based on protein sequences annotated from the assembled whole genomes showing the relationship of *Streptomyces antibioticus* SCBA4 (highlighted in blue) with closely related species. Bootstrap values are shown as percentages of 1000 replications. Bar shows number of amino acid substitutions per site.

Out of the 573,450 aligned sites, 194,699 (33%) were variable, 343,090 were conserved, 61,298 were singletons and 130,267 were parsimony informative sites. *S. antibioticus* SCBA4 is very closely related to *Streptomyces antibioticus* DSM 41481 (CP050692.1). Reference mapping with *S. antibioticus* DSM 41481 produced 95.1% pairwise identity with 7,150,628 identical sites indicating their close relationship and high genetic similarity. Digital DNA-DNA hybridization (dDDH) analysis yielded values of 83.7% (Formula 1), 67.6% (Formula 2), and 83.6% (Formula 3). Average Nucleotide Identity (ANI) value is 96.17%. The dDDH results, together with the high ANI value and phylogenetic analysis confirm taxonomic identity. The next closest relative is *Streptomyces longwoodensis* NBC 00642 (CP130729.1). The generated phylogenetic tree also shows that *Streptomyces* species forms a single clade that is distinct from *Kitasatospora* species, a closely related genus (Fig. 2).

## 4 Experimental Design, Materials and Methods

### 4.1 Isolation of actinobacteria

*Streptomyces antibioticus* SCBA4 was isolated from the rhizosphere of *Momordica charantia* L. (bitter gourd) planted in 10 m^2^ plot of soil in an organic farm complex at the City Organic Agriculture Fisheries Complex (COAFC) in Bislig, Surigao del Sur, Philippines (8°15′25.2"N 126°17′56.0"E). Sampling was performed in February 2019 and local climate was a combination of wet and dry season. At least five spot samples were taken, resulting in a total of 1 kg of pooled soil sample for every 10 m^2^ plot. Selective pre-treatment of the samples by air drying for 2 days was first done to reduce the numbers of non-spore-forming bacteria. Ten (10) grams of air-dried soil samples were suspended in 90 ml of 0.85% NaCl and serially diluted into Yeast Malt Agar (yeast extract 10 g/L; malt extract 25 g/L; glucose 10g/L) with 50 mg/L nystatin. The plates were incubated at 28°C for 3-7 days. Colonies that appeared as brown or white or creamy-white to moderate yellow-orange, hard colonies on YMA [6] and have a distinct characteristic of actinobacteria were selected. Isolated colonies were purified on YMA and stored in 60% glycerol stocks at -20°C for storage. *Streptomyces antibioticus* SCBA4 exhibited antimicrobial activity against methicillin-resistant *Staphylococcus aureus* (MRSA) during preliminary trials and was hence selected for further genomic studies.

### 4.2 Preliminary testing for antimicrobial activity against methicillin-resistant *Staphylococcus aureus*

Preliminary antimicrobial testing was performed against methicillin-resistant *Staphylococcus* (MRSA) BIOTECH 10378. The test isolate was first incubated in Mueller Hinton (MH) broth at 37°C for 24 hours. The cells were harvested via centrifugation, resuspended and diluted to 0.5 MacFarland standard before inoculating into pre-poured MH agar plates using sterile cotton swab. Agar plugs (6 mm) of *S. antibioticus* SCBA4 (3 days old in YMA plate at 28°C) were cut using a sterile borer and placed face-down into the surface of the medium seeded with the test organism. The plates were incubated at 37°C for 24 hours and the zone of inhibition was measured using a digital caliper [7]. Vancomycin disks were used as positive control.

### 4.3 Genomic DNA Isolation, whole genome sequencing, assembly, and annotation

The isolates were grown in Tryptic Soy Broth (TSB) for two days and the genomic DNA was extracted using the ZR Fungal/Bacterial DNA MiniPrep Kit (Zymo Research, Irvine, CA) following manufacturer’s instructions. Extracted gDNA was sent to Macrogen (South Korea) for de novo whole genome sequencing. Library preparation was done using using TruSeq Nano DNA Library (350bp insert) kit and sequencing using NovaSeq6000 platform with 150-bp paired-end setting. A total of 22,095,266 150-bp paired end reads were generated, corresponding to an estimated genome coverage of approximately 387x. Raw sequencing reads were of high quality, with 96.7% and 92.0% of bases achieving Phred quality scores of Q20 and Q30 respectively. Adapter and low-quality sequences were removed using Trim Galore version 0.6.7 [8], resulting in the trimming of 0.4% of the total sequenced bases prior to genome assembly. QC reads are then assembled de novo using SPAdes V3.13.0 [9] and polished using Pilon version 1.23 [10]⁠, implemented in Unicycler 0.5⁠. De Novo assembled contigs [11]. Assembly quality was assessed with BUSCO v5.8.3 [12] using the Streptomyces_odb12 database. No plasmid-like contigs were identified during genome assembly. Gene calling and annotation is done using Prokka V1.14.6 [13] through GALAXY Europe (usegalaxy.eu). The presence of biosynthetic gene clusters was determined using antiSMASH [14] webserver (version 8.0) with relaxed detection strictness and turning on all the extra features. Carbohydrate-active enzymes (CAZymes) were identified by submitting the assembled sequence to the dbCAN3 web server (https://pro.unl.edu/dbCAN2/blast.php). CAZyme annotations supported by at least two of the three integrated tools (HMMER, DIAMOND, and dbCAN0sub) were retained and classified into the corresponding CAZy families.

### 4.4 Phylogenetic tree construction

To infer the phylogenetic placement of *Streptomyces antibioticus* SCBA4, type strains were selected based on the closest relatives identified by genomic BLAST searches together with representative *Streptomyces* species selected from previously published *Streptomyces* phylogenies. Two (2) *Kitasatospora* species were designated as outgroup since it is the closest phylogenetic relative of *Streptomyces* while still remaining a distinct genus and is widely used for rooting *Streptomyces* phylogenies [[15], [16]]. The complete genome sequences of all six *Streptomyc*es and two *Kitasatospora* species were retrieved from GenBank. Protein BUSCO analysis was performed for each assembly, and the recovered complete single-copy BUSCO protein sequences were extracted. Orthologous BUSCO proteins shared among all accessions were concatenated and, subsequently, aligned using MAFFT v7.4 [17]. Phylogenetic reconstruction was performed using the maximum likelihood method using IQ-TREE v1.6.9 [18] with the best model (LG+G+I+F) and ultrafast bootstrapping (-bb 1000) [19]. The assembled sequences were reference mapped to the closest relative using BBMap 38.84 in Geneious Prime 2026.0.2 [20]. Additionally, average nucleotide identity (ANI) and digital DNA-DNA hybridization (dDDH) analyses were performed using the ANI Calculator available in EZBioCloud (https://www.ezbiocloud.net/tools/ani) and the Genome-to-Genome Distance Calculator (GGDC), respectively [21].

## Limitations

Not applicable.

## Ethics Statement

The authors have read and followed the ethical requirements for publication. The study does not involve human subjects, animal experiments nor data collected from social media platforms.

## CRediT Author Statement

**Cherray Gabrielle A. Macabecha:** Methodology, Formal Analysis, Validation, Investigation, Data Curation, Writing – Original Draft, Visualization; **Irene A. Papa:** Conceptualization, Resources, Writing – Review and Editing, Supervision, Project Administration, Funding acquisition.
